# Cognitive and behavioural strategies for self‐directed weight loss: systematic review of qualitative studies*

**DOI:** 10.1111/obr.12500

**Published:** 2017-01-24

**Authors:** J. Hartmann‐Boyce, A.‐M. Boylan, S. A. Jebb, B. Fletcher, P. Aveyard

**Affiliations:** ^1^ Nuffield Department of Primary Care Health SciencesUniversity of OxfordOxfordUK

**Keywords:** Qualitative, self‐management, systematic review, weight loss

## Abstract

**Aim:**

We conducted a systematic review of qualitative studies to examine the strategies people employ as part of self‐directed weight loss attempts, map these to an existing behaviour change taxonomy and explore attitudes and beliefs surrounding these strategies.

**Methods:**

Seven electronic databases were searched in December 2015 for qualitative studies in overweight and obese adults attempting to lose weight through behaviour change. We were interested in strategies used by participants in self‐directed efforts to lose weight. Two reviewers extracted data from included studies. Thematic and narrative synthesis techniques were used.

**Results:**

Thirty one studies, representing over 1,000 participants, were included. Quality of the included studies was mixed. The most commonly covered types of strategies were restrictions, self‐monitoring, scheduling, professional support and weight management aids. With the exception of scheduling, for which participant experiences were predominantly positive, participants' attitudes and beliefs surrounding implementation of these groups of strategies were mixed. Two new groups of strategies were added to the existing taxonomy: reframing and self‐experimentation.

**Conclusions:**

This review demonstrates that at present, interventions targeting individuals engaged in self‐management of weight do not necessarily reflect lived experiences of self‐directed weight loss.

## Introduction

Overweight and obesity are a major cause of preventable morbidity and mortality worldwide, with the World Health Organization estimating that they cause at least 35.8 million disability adjusted life years and 2.8 million deaths annually 1. For these reasons and others, many adults try to lose weight: at any one time, over a quarter of American women are trying to lose weight (27%), with men not far behind (22%) 2. The large majority of adults trying to lose weight are doing so without professional input or a formal weight loss programme. However, in contrast to more intensive interventions 3, 4, 5, very little is known about self‐directed efforts to lose weight.

It is widely recognized and accepted that increasing energy expenditure and decreasing energy intake (in effect, creating a negative energy balance) lead to weight loss in otherwise healthy adults. However, despite this seemingly simple formula, weight loss efforts are often unsuccessful, and in adults who manage to initially lose weight, weight regain is common, due in part to powerful biological and environmental forces. Therefore, the issue may not be *what* changes to make to diet and physical activity, but *how* to ensure people manage to make these changes and sustain them in the long term. Current research focuses on how poor diet and lack of activity cause disease 6, 7, but we know much less about how changes in these behaviours can be initiated and maintained – in particular, very little is known about the cognitive and behavioural strategies that influence these behaviours, which can include elements such as self‐monitoring, strategies to boost motivation and social support, among others.

As a first step in developing further understanding of this area, we created a taxonomy of these cognitive and behavioural strategies, called the Oxford Food and Activity Behaviours (OxFAB) taxonomy 8. To date, this has been used to categorise the content of self‐help interventions for weight loss as part of a quantitative systematic review and meta‐analysis, and has been translated into a questionnaire and used in a cohort study to examine the relationship between use of these strategies and weight change trajectories in British adults trying to lose weight 9, 10. To our knowledge, no systematic reviews currently review qualitative evidence specific to self‐directed weight loss and weight loss maintenance. This review of qualitative literature is therefore a crucial further component to understanding the cognitive and behavioural strategies used by overweight and obese adults in weight loss attempts. The review aims to:

- examine the strategies people employ as part of self‐directed weight loss attempts;
- test the current version of the OxFAB taxonomy against narrative descriptions of weight loss, refining and adding new terms if warranted; and
- explore attitudes and beliefs surrounding the implementation of these strategies as part of self‐directed efforts to lose weight.

## Methods

Details of the protocol for this systematic review were registered on PROSPERO prior to work commencing 11.

### Search

Seven electronic databases were systematically searched in December 2015 (CINAHL, EMBASE, MEDLINE, PsycINFO, Science Citation Index Expanded, Social Science Citation Index, Conference Proceedings Citation Index – Science) for qualitative studies using terms related to qualitative research methodologies, obesity, weight loss, diet, exercise, behaviour change and self‐care. Search terms for obesity, behaviour change and self‐care were adapted from a recent systematic review of self‐help interventions for weight loss, 8 and search terms relating to qualitative methodology are those proposed by the Cochrane Collaboration 12. MEDLINE search terms are listed in full on PROSPERO 11. Reference lists of included studies and relevant systematic reviews were also screened for further studies.

### Inclusion criteria

The SPICE framework (settings, participants, interest, comparison, evaluation) was used to define inclusion criteria 13. Settings included community and primary care, and participants included adults (18 or older) who had attempted or were attempting to lose weight through behaviour change. Studies exclusively in people with anorexia nervosa or bulimia nervosa were excluded. The interest was those strategies used by participants in self‐directed efforts to lose weight, defined as identifiable and unique behaviours or cognitions designed to help participants achieve weight‐loss targets or adhere to diet or physical activity targets explicitly undertaken in an effort to lose weight. We did not extract other outcomes and did not include studies evaluating participants' experiences with, or opinions of, specific weight loss interventions (e.g. programme evaluations), as the aim of this review was to focus exclusively on self‐management, including those strategies used by individuals that may not be advised by standard self‐help information or be deemed acceptable in a trial context. We did not restrict studies on the basis of comparisons, and included only qualitative studies, e.g. interview, semi‐structured interview, open‐ended surveys and focus groups. Non‐English language articles were excluded. There were no restrictions on publication date or country.

### Screening and data extraction

One reviewer screened titles and abstracts for inclusion, with a sample of 10% checked by a second reviewer. The agreement rate was 100%. Full text was screened by one reviewer. Data extraction was conducted independently by two reviewers for all included studies using an adapted version of the QARI (qualitative report data extraction) form developed by the Joanna Briggs Institute for Evidence Based Practice 14. The form was piloted before use by two reviewers and amended as necessary. Data extraction consisted of three main components: study characteristics (including research aims, methods, setting and participant details), quality assessment (using the Critical Appraisal Skills Program [CASP] for qualitative studies 15) and self‐management strategies. Discrepancies were resolved by discussion or, where necessary, through referral to a third reviewer.

Self‐management strategies were extracted using a modified framework approach 16, 17. Two reviewers independently identified cognitive and behavioural strategies for weight loss in the included studies and coded these against a checklist of previously identified domains of strategies 8. Where present, reviewers also extracted data relating to use of self‐management strategies more generally, or relating to cognitive and behavioural strategies for weight loss which were not included in the first version of the OxFAB taxonomy (Table 1).

**Table 1:** OxFAB taxonomy domains and definitions

Domain	Definition
Energy compensation	Conscious adjustment of behaviours to alter energy intake and/or expenditure to control weight in light of previous energy intake or expenditure
Goal setting	Setting of specific behavioural or outcome target(s)
Imitation (modelling)	Emulating the physical activity or dieting behaviour of someone who you have observed
Impulse management: Acceptance	Respond to unwanted impulses through awareness and acceptance of the feeling that generates the impulse and reacting without distress or over‐analysis
Impulse management: Awareness of motives	Respond to unwanted impulses by evaluating personal motives behind that impulse before acting
Impulse management: Distraction	Respond to unwanted impulses through distraction in an attempt not to act on the impulse
Information seeking	Seek specific information to enhance knowledge to help manage weight
Motivation	Strategies to increase the desire to control weight
Planning content	Plan types of food/physical activity in advance of performing behaviour
Scheduling of diet and activity	Plan timing and context/location of food/physical activity in advance of performing behaviour
Regulation: Allowances	Unrestricted consumption of or access to pre‐specified foods or behaviours
Regulation: Restrictions	Avoid or restrict pre‐specified foods, behaviours or settings
Regulation: Rule setting	Mandate responses to specific situations
Restraint	Conscious restriction over the amount that is eaten
Reward	Reinforcement of achievement of specific behaviour or outcome through reward contingent on the meeting of that target
Self‐monitoring	Record specific behaviours or outcomes on regular basis
Stimulus control	Alter personal environment such that it is more supportive of target behaviours (adapted from CALO‐RE) 18
Support: Buddying	Perform target behaviours with another person
Support: Motivational	Discussing, pledging, or revealing weight loss goals, plans, or achievements or challenges to others to bolster motivation
Support: Professional	Seek help to manage weight from someone with specific expertise
Weight management aids	Use of and/or purchase of aids to achieve weight loss in any other manner (includes ingested agents such as medications, over‐the‐counter products and supplements; also includes exercise equipment)

### Analysis

Verbatim text on self‐management strategies was coded using NVivo 11 19. This included both direct quotes from participants as well as authors' summaries and interpretations of data. Where studies yielded strategies that had not yet been identified in the taxonomy, these were used to expand the framework through the addition of new index terms and/or top level categorizations (domains). This analysis was based on the principles of the thematic synthesis approach, set forth by Thomas and Hardern 20 and detailed by Major and Savill‐Baden. 21 Thematic synthesis draws on the methods used in thematic analysis of primary sources, extending them for use in systematic reviews and consists of three analytical steps: identifying and analysing first order themes (through line by line coding), synthesising second order themes (through organizing free codes into related areas to construct descriptive themes) and interpretation of third order themes (the development of analytical themes). Two reviewers independently inductively and deductively coded data on self‐management strategies. In instances where it was unclear how to code strategies against the initial framework, the strategies were discussed in consultation with a further two reviewers to reach consensus on whether a new domain should be formed or whether an existing domain should be expanded. Findings are synthesised narratively.

## Results

### Search results

Excluding duplicates, searches yielded 2,284 references (Fig. S1). After full text screening, 36 references, representing 31 studies, were included. Of these, six were unpublished theses. The most common reason for exclusion at full text stage was that the study was an evaluation of a specific weight loss programme, rather than focussed on self‐directed weight management efforts.

### Characteristics of included studies

Details on key individual study characteristics can be found in Table 2. An overview is provided in the succeeding texts.

**Table 2:** Characteristics of included studies

Study ID	Country	Focus	Inclusion criteria	N	Mean age	% female	mean BMI	SES	Ethnicity
Abolhassani, 2012 31	Iran	Barriers and facilitators to weight gain and loss	Unsatisfied with current weight, tried to reduce weight at least once. Excl. lack of interest, dialect/language differences, limitations and inability to speak	11	NS	NS	NS	Eight employed, no other detail provided	NS
Ali, 2010 32	United Arab Emirates	Weight management behaviours and perceptions of women at increased risk of type 2 diabetes within UAE cultural context	Emirati national women, 18 years old or older, no previous diagnosis of diabetes (except gestational), with one or more of the following: gestational diabetes, abdominal obesity (weight circumference >88 cm) + family history of type 2 diabetes, or prediabetes (fasting plasma glucose or glucose load test)	75	39	100	NS	NS	NS
Allan, 1991 33	USA	Weight management in white women	Normal weight to moderate obesity (40–100% over ideal weight); born in US and living in study area; 18–55 years old; White	37	33.7	100	NS	57% middle class, 43% working class; all but three is employed. 30% high school grad, 32% some college, 38% college grad	White
Barnes, 2007 23	USA	Weight loss maintenance as it relates to the theory of planned behaviour	African–American women, ≥18, lost ≥10% of body weight and either regained or maintained for a year	37	41.6	100	32.75	84% employed; Highest level of education: High school 22% regainers (R), 0 maintainers (M); Some college 29% M; 48% R; College grad 50% M; 22% R; Grad school 21% M; 8% R	African–American
Befort, 2008 22	USA	Perceptions and beliefs about body size, weight and weight loss among obese African–American women	≥18, African–American, female, obese according to self‐reported weight and height. Excl. obvious intoxication or current inpatient for substance abuse treatment, marked inappropriate affect or behaviour, acute illness or impaired cognition	62	46.6	100	40.3	15% some high school, 21% HS grad; 63% some college, 2% college grad; 50% full time employed; 8% part time; 42% not employed	African–American
Bennett, 2013 34	UK	How men communicate with each other about their bodies, weight management projects and masculinities	NS	116	NS	0	NS	NS	NS
Bidgood, 2005 35	UK	Obese adults' experiences and feelings about weight loss attempts and maintenance	Obese men and women ≥18, BMI ≥30	18	NS	89	NS	NS	NS
Byrne, 2003 36	UK	Psychological factors associated with successful and unsuccessful weight maintenance	Female, aged 20–60 years, history of BMI >29.9 who at some point in last 2 years lost ≥10% weight through deliberate caloric restriction. Maintainers: maintained lower weight (within 3.2 kg) for ≥1 year. Regainers: Regained to within 3.2 kg of original weight. Excl. weight loss due to medical/ psychiatric condition or use of medication; weight loss or regain because of pregnancy or childbirth, history of anorexia or bulimia	56	41	100	NS	Social class 1–2 47%; 3 nm–3 m 30%, 4–5 1%; students 13%; housewives 7%; unemployed 1%	NS
Callen, 2008 37	USA	Weight change in older adults, focussing on methods	Community dwelling, ≥80, ‘cognitively intact or mild intellectual impairment’, English speaking, BMI ≥27, able to stand for height and weight	9	82	33	30.17	Education range 8th grade to postgrad. Two had incomes below poverty level	NS (‘lack of ethnic representation’)
Chambers, 2012 38	UK	Long term weight maintenance	30 years or older, wide range of weight experiences. Excl factors that could impact directly on current weight (incl. pregnancy, some medications, medical conditions, and anorexia)	14	48	75	NS	NS	Caucasian
Chang, 2008 39	USA	Motivators and barriers to healthful eating and physical activity among low‐income overweight/obese non‐Hispanic black and white mothers	Women, non‐Hispanic white or non‐Hispanic Black, 18–35 years old, not pregnant or breastfeeding, able to speak and read English, BMI 25–39.9, interested in prevention of weight gain, ≥3 months postpartum, ≥1 child enrolled in government food and nutrition service programme	80	25.8	100	31.15	47% high school or less education	41 non‐Hispanic black; 39 non‐Hispanic white
Collins, 2012 25	USA	Perceptions of previously obese individuals after self‐guided weight loss	Female, aged 35–60, self‐identified as ‘obese‐reduced weight maintainers’ of ≥10% of original weight for ≥1 year	11	45.6	100	NS	NS	NS
Davis, 2014 26	USA	Experiences of college students in the weight‐loss process	Full time students at one Midwestern university considered overweight at some point during college enrolment, active in trying to lose weight for ≥6 months, willing to be interviewed, 18 years or older	5	NS	60	NS	NS	Four Caucasian; one ‘person of colour’
Diaz, 2007 24	USA	Weight loss experiences, attitudes and barriers in overweight Latino adults	Age ≥20, BMI ≥25, self‐identified Latino	21	NS	90	NS	Five had education beyond high school	Self‐identified Latinos
Faw, 2014 40	USA	Support management strategies used by overweight young adults attempting to lose weight	Perceive themselves as being overweight or obese, attempted to lose weight at least once during past year (all undergraduate university students)	25	21.1	64	27.1	NS	Asian/ Asian American 44%; white 40%
Frank, 2012 27	USA	Weight loss maintenance	History of weight cycling; highest ever BMI≥30; maintained loss reflects BMI of 18.5–24.9; weight loss achieved without bariatric surgery and maintained for ≥3 years; American born and raised	10	NS	90	NS	Two some college; Three completed college; Five college + advanced degree	Eight Caucasian, one Latina and one biracial
Green, 2009 41	UK	Phenomenology of repeated diet failure	Over 18, speak fluent English, ≥2 serious attempts to diet which they considered had failed, unhappy with current eating habits. Excl eating disorder or medical/psychological input re: eating	11	40	82	NS	NS	One British Pakistani; 10 white British
Heading, 2008 42	Australia	Risk logics, embodiment, issues related to adult obesity in remote New South Wales	‘rural adults’, ‘history of unwanted weight’	19	NS	68	NS	Education ranged from some high school to postgraduate qualifications	NS
Hindle, 2011 43	UK	Experiences, perceptions and feelings of weight loss maintainers	Maintained ≥10% weight loss for ≥1 year, stable weight for last 6 months, 18 years or older and English speaking. Excl. weight loss through bariatric surgery, VLCD, within 6m of giving birth	10	44	100	25.8	‘Employed, retired or housewives with employed partners’	Caucasian
Hwang, 2010 44	USA	Social support for weight loss in web community	Members of SparkPeople.com online weight loss community	13	36	100	NS	NS	White
Jaksa, 2011 28	USA	Experience of maintaining substantial weight loss	Maintained weight loss for ≥2 years; lost ≥20% body weight; within 10–15 lb of their goal weight; willing to commit to reflecting on their experience through the process of an audiorecorded interview; not undergone any surgical procedures affecting or manipulating appetite regulation; at least 20 years old	12	NS	92	NS	Four graduate students; five full time employed; one part time employed; one stay at home mother; one on long term disability	NS
Karfopoulou, 2013 45	Greece	Weight loss maintenance and Mediterranean diets	20–65 years old, at some point in their lives BMI >25 (excl. pregnancy), intentionally lost ≥10% of starting weight. Maintainers had to be at or below the 10% weight loss for ≥1 year, regainers had to be at a weight ≥95% of their starting weight. Excl. history of anorexia	44	33	59	27.65	NS	NS
Macchi, 2007 29	USA	Process of meaning‐making associated with weight loss and maintenance	Female, 30–45 when initially lost weight, intentionally lost ≥10% of initial body weight without undergoing bariatric surgery and maintained ≥10% lost	10	NS	100	NS	NS	All white
McKee, 2013 46	UK	Weight maintenance	Previous BMI ≥25, intentionally lost 10% through diet and/or exercise and maintained for ≥12 months within range of 2.2 kg OR regained weight lost	18	45	89	28.3	Non‐academic university staff, self‐employed or retired members of the public	10 British, 5 South Asian, 3 other
Reyes, 2012 47	USA	Weight loss maintenance	25–64 years old, intentionally lost ≥10% weight in past 2 years; regainers regained ≥33% of their weight loss and maintainers regained ≤15%. Excl participants with type 2 diabetes, history of cancer, or bariatric surgery	29	47	65.6	32.5	NS	41% white; 59% African–American
Sanford, 2012 48	US, UK, Canada	Weight loss blogs	Need to lose ≥100 lb (not clear how this was defined), had been blogging for ≥3 months about weight loss. Excl bariatric or lap band surgery	50	40	80	NS	NS	NS
Stuckey, 2011 49	USA	Successful weight loss maintenance practices	lost ≥30 lb and maintained for ≥1 year, age >21, not pregnant, English speaking. Excl. bariatric surgery	61	NS	72	NS	90% at least some college	79% white
Su, 2015 50	Taiwan	Taiwanese perimenopausal women's weight loss experience	Women 45–60 years, undergoing perimenopause (self‐report); BMI ≥27; trying to lose weight; could communicate in Mandarin and Taiwanese; met diagnostic criteria for metabolic syndrome for Asian populations (e.g. >3 of (1) waist circumference ≥80 cm, (2) fasting blood glucose ≥100 mg dl^−1^, (3) high‐density cholesterol <50 mg dl^−1^, (4) triglycerides ≥150 mg dl^−1^ (5) systolic pressure ≥130 mmHg or diastolic ≥85 mmHg)	18	52	100	32.6	5 housewives, 13 employed. 7 had attended university.	NS
Thomas, 2008 51	Australia	Lived experiences of obesity and weight loss attempts	BMI ≥30	76	47	83	42.5	51% unemployed. 45% at least completed high school	80% White Australian; 5% English; 20% Other European
Tyler, 1997 52	USA	Weight loss methods among women	Female, 18–60 years without major health problems, not pregnant, US born, living in study area, normal or overweight BMI	80	34	100	NS	50% higher SES (Hollingshead index 40‐66); 50% lower SES (8‐39). 26 high school or less; 28 partial college; 13 college graduate; 12 graduate degree	40 African–American and 40 Euro American
Witwer, 2014 30	USA	Weight loss maintenance	Adult (18 years or older), lost ≥10% of body weight and maintained loss for ≥1 year, excl. bariatric surgery, unintentional weight loss, residents of long‐term care settings, non‐English speakers	12	NS	66	NS	3 some college, 9 college degree; 9 full time employed, 2 part time, 1 retired	NS

*Note*: NS=not specified; Excl=excluded; SES: socio economic status

### Methods

Of the 31 included studies, seven used focus groups and 22 used one‐to‐one interviews, alone or in combination with other methods. The final two studies used web content as the basis for analysis: one collected data from a web forum on weight loss linked to a popular male magazine and the other collected data from a weight loss blogging website, and also administered qualitative surveys to bloggers. In terms of methods for analysing data, six used a form of phenomenological analysis, six used thematic analysis and eight reported using grounded theory. The remainder did not report their approach.

Twelve of the studies did not report their sampling methods; of those that did, the most common methods (in order of frequency) were purposive sampling (nine studies), convenience sampling (four studies), theoretical sampling (three studies), and snowball, random, and maximum variation sampling (one study each). Where reported, recruitment was primarily through advertisements in local media, flyers in public places (some targeting gyms and locations where weight loss programmes were offered), and by word of mouth and through personal contacts. Two studies posted flyers in medical centres, and one recruited via referrals from a health and wellness centre.

### Participants

Combined, the included studies represent 1,050 participants. The majority of studies 17 took place in the USA. Ten studies focussed exclusively on weight loss, and eleven focussed exclusively on weight loss maintenance. Of the latter, five explicitly recruited ‘regainers’ and ‘maintainers’, and focussed on differences between the two. Seven focussed exclusively on experiences within particular population groups, e.g. by ethnicity or age range.

Across the 21 studies that reported it, the average age of participants was 42. Studies predominantly contained more women than men. Where reported (11 studies), average BMI across the studies was 31.9 kg m^−2^, ranging from 25.8 (study of successful, previously obese weight loss maintainers) to 42.5 kg m^−2^. Of the 18 studies that reported data on ethnicity, 11 represented all or majority white populations. Two included only African–American participants 22, 23, and one included only Latinos 24. Approximately half of the studies reported data relevant to socioeconomic status; of these, the majority reported including predominantly well‐educated and middle to high socioeconomic status participants.

### Quality of included studies

Quality of included studies was mixed, in part reflecting that a proportion of the included studies had not been published in peer reviewed journals (unpublished doctoral theses) 25, 26, 27, 28, 29, 30. A summary of answers for each CASP domain is presented in Table 3. Issues were predominantly related to recruitment methods, the relationship between the researcher and participants, and provision of sufficient detail on the method of analysis.

**Table 3:** Summary of quality judgements

Critical Appraisal Skills Program question	Number of answers across all included studies		
	Yes	Unclear	No
Was there a clear statement of the aims of the research?	29	0	2
Is a qualitative methodology appropriate?	30	1	0
Was the research design appropriate to address the aims of the research?	26	3	2
Was the recruitment strategy appropriate to the aims of the research?	13	10	8
Was the data collected in a way that addressed the research issue?	25	5	1
Has the relationship between researcher and participants been adequately considered?	5	18	8
Have ethical issues been taken into consideration?	15	14	2
Was the data analysis sufficiently rigorous?	12	10	9
Is there a clear statement of findings?	16	10	5
Is the research valuable?	24	6	1

### Cognitive and behavioural strategies

#### Strategies employed in weight loss attempts

**Figure 1:**
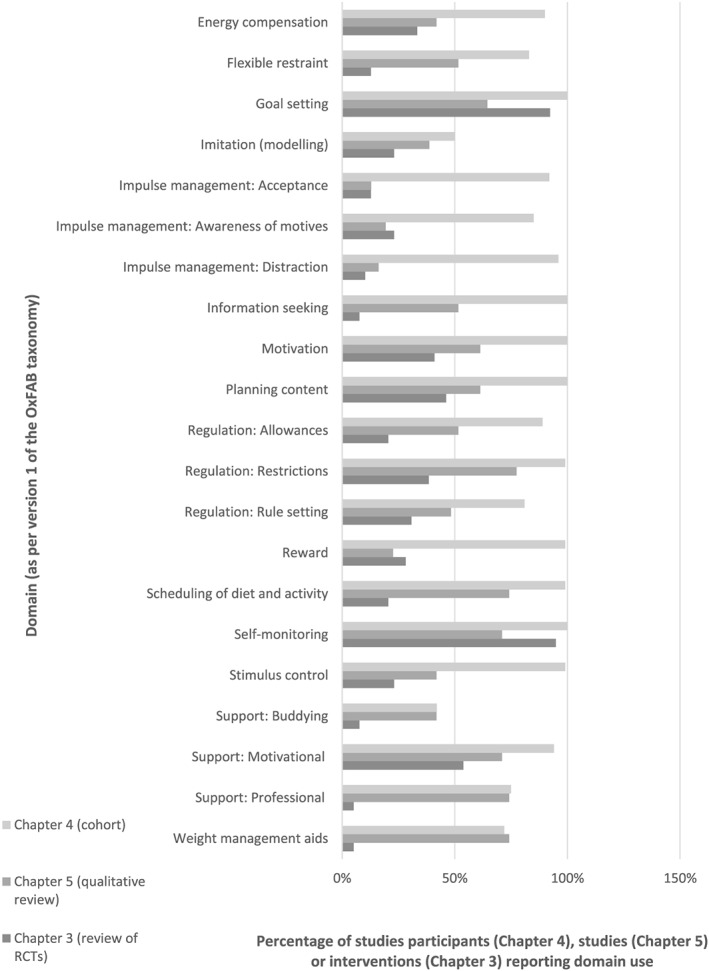
Frequency of domain coding across included studies (using OxFAB taxonomy), compared with domain coding from separate review of self‐help interventions 9. *Note*: * new domain introduced through process of this review. As such, these domains are new to this review and hence were not used to code self‐help interventions.

The most commonly discussed groups of strategies were restrictions, scheduling of diet and activity, self‐monitoring, professional support and use of weight management aids (Fig. 1). Generally, and in part reflecting the varied interests of the studies, there was little information on attitudes and beliefs regarding implementation of these strategies. Where attitudes and beliefs around specific strategies were discussed, these are reported in the succeeding texts. A separate section (‘Implementation of strategies’) discusses findings relating to participant choice and use of strategies more broadly.

For each of the most commonly discussed groups of strategies, content predominantly related directly to dietary change. For restrictions, this included avoiding certain foods, particularly high fat, high calorie and high sugar items. Participants spoke of cutting out specific foods, rather than groups of foods (e.g. gravy 33 and wine 38). They also mentioned meal skipping and portion control methods, and spoke of avoiding certain settings as a way to restrict access to food, including restaurants and family gatherings. 28, 30, 40 Negative attitudes were expressed in relation to restrictions, with participants expressing feelings of deprivation. These feelings were presented as challenges to maintaining use of these strategies, as feelings of deprivation could lead to participants ‘falling off the wagon’. 27, 43, 46, 47 In regard to scheduling, the most commonly occurring strategies related to scheduling meals (e.g. three meals a day with no snacking outside of meal times) 30, 33, 45, 49 and the practice of not eating late at night or only eating specific foods after a certain time in the evening. 50, 52 Studies also reported participants' efforts to schedule physical activity at a time that fit with their lifestyles and preferences, including exercising at times where fewer people were present to avoid embarrassment 35. One study conducted exclusively with African–American women reported on the importance of scheduling time for hairstyle maintenance after exercise 23. Generally, strategies in this domain were discussed in a positive manner, with scheduling viewed as a way in which to establish a sustainable routine 25, 26. For example, one participant explained, ‘You just gotta get into that schedule. And its automatic and it just really makes it easier when I do have a routine. If I don't have a routine, God knows I don't have an idea what things would look like, because it would just be so sporadic.’ 25

Self‐monitoring strategies most commonly focussed on self‐weighing and monitoring food intake, specifically calories. Participants also spoke of monitoring fitness, either in terms of time, distance, steps or calories burnt. In addition to weighing themselves, participants discussed other ways of monitoring their weight, including visual inspection in a mirror 33, 34, 36, 45, the fit of clothing 23, 33, 38, 42 and physical capabilities (e.g. climbing stairs and reaching one's toes) 25, 38, 47. Attitudes and beliefs surrounding self‐monitoring were mixed and often strongly expressed. Negative aspects included difficulties with maintaining vigilance over the long term and feelings of shame related to food consumption and weight 8, 25, 35. In two studies, participants cited fear as a barrier to continued self‐weighing. 36, 47 In contrast, more positive takes on self‐monitoring included assertions that it led to increased feelings of self‐efficacy and self‐control, as well as increased accountability for one's own actions 28, 43, 46, 48; in one study, a participant went so far as to call the weighing scale his ‘best friend’. 47

Finally, use of professional support and weight management aids occurred in many participant narratives, again accompanied by mixed attitudes and beliefs. Studies often included participants who had formerly attended weight loss programmes, and those who had solicited help from personal trainers, doctors and nutritionists. Negative experiences included advice that did not fit with participants' daily routines 31, 32, experiences of relapse once programmes ended 25, 46 and the financial costs of accessing such support 26, 47, 51. Positives included motivational support and accountability 26, 43 and access to trusted information 24, 26. The latter particularly pertained to personal trainers who helped with exercise regimes. The weight management aids discussed included medications, over‐the‐counter supplements, exercise equipment and exercise videos. In a study of Emirati women, participants spoke positively of these aids as a way to overcome cultural barriers to weight loss, which included cultural norms surrounding physical activity outside of the home and dietary constraints involving cooking for guests 32. Other studies noted negative views towards weight loss medications specifically, with participants referring to them as ‘unnatural’ 22, 25 and expressing concerns about side effects and weight regain once medication was discontinued 51. In one study, participants referred to weight loss medications as ‘band‐aids’, implying that they were a temporary fix to a problem requiring greater intervention 25.

#### Mapping and expansion of OxFAB taxonomy

All OxFAB domains were covered in multiple publications, ranging from three (impulse management domains) to 24 times (regulation: restrictions) (Fig. 1). Strategies not covered in the first version of the OxFAB taxonomy also emerged. This led to the introduction of two new domains, namely reframing and self‐experimentation. Self‐experimentation, a recognized technique in behaviour change interventions, refers to the process of experimenting with different techniques and behaviours, assessing their outcomes and deciding whether or not to continue based on the observed outcome 53. Studies described this as the mechanism by which participants chose a ‘primary strategy’ to use in a weight loss attempt 25, 33, using ‘self‐analysis’ to create eating and exercise plans 29, 52. No studies discussed participants' attitudes or beliefs about use of this strategy.

Reframing refers to the process of redefining the behaviours and process of weight loss, shifting from ‘diet’ terminology to thinking about weight loss behaviours as ‘a way of life.’ 26, 27, 29, 30, 45 This included participant statements such as: ‘It's not a diet … . I try hardly ever to say that word. … Because it's gotta be lifestyle’ 27; ‘I went with the belief that this wasn't a diet, but what I'd got to do was change my way of eating’ 42; and, ‘you've got to tell yourself you're not on a diet you're just changing your way of life.’ 46 In other studies, participants used specific metaphors as a way of reframing, re‐envisaging food as ‘fuel’, ‘drugs’ or ‘poison’ 28, and hunger pangs as ‘Pac Men [video game animations] eating away… at fat’ 49. Participants who described using reframing strategies spoke of their positive role in increasing long‐term commitment to their weight management practices and boosting their self‐esteem 27, 42, 43, 45. However, not all discussions of reframing were positive: one participant found it ‘hard’ to reframe food as a ‘vice’, as she'd previously thought of it as a ‘comfort item’ that was now no longer available to her 29.

In addition to the previously mentioned new domains, impulse management domains were expanded to include delay (responding to an unwanted impulse by delaying the desired action 28, 50, 52) and substitution (using a physical substitution for eating, e.g. chewing on a toothpick 24, 28, 30, 45, 49, 52). Finally, a new weight management aid was also identified, namely a girdle, highlighting the existence of some weight control practices that are culture‐specific. In this study of Latino adults, the authors explain that, although discouraged in the US, using a girdle post‐partum is considered an effective weight management technique in Mexico 24.

#### Implementation of strategies

In addition to covering specific strategies, there was also some reflection on the ways in which participants selected and implemented the strategies they would use, although generally this was limited and related more to the selection of strategies as they related to one another or stages in weight loss attempts, rather than to ways in which attitudes and beliefs influenced these choices. In a study exploring differences between people who regained weight lost versus those who maintained their initial weight loss, the authors state that maintainers spoke of having a number of strategies they could employ when seeking to manage their weight, and contrasted this with regainers who usually attempted to lose weight ‘via a single strategy of reducing their calorie intake’. 38 A second study found that although participants experimented with a number of different strategies for weight loss, they usually had a preferred method that they repeatedly turned to. The most common ‘primary weight loss methods’ identified by the authors were reducing high calorie foods, increasing the intake of low calorie food and exercising on one's own 52.

Other studies reflected how strategy choices related to one another and changed over time. Faw (2014) focussed exclusively on methods relating to social support and found clusters of strategies, with some participants favouring direct approaches (e.g. directly soliciting support, confronting those who did not offer it) and others using a variety of indirect methods (e.g. complaining as a way to elicit support, avoiding people who did not offer support). The author labels this the ‘direct/indirect strategy continuum’ 40. Collins (2012) found that the strategies selected by participants were ‘unique’ depending on the participant and changed over time through the process of self‐experimentation 25. Allan (1991) divided the weight management process into stages (appraising, de‐emphasising, mobilising, enacting and maintaining) and noted that each stage consisted of multiple processes that were characterised by the use of specific tactics or strategies. The complexity of these strategies and tactics increased with each stage of the process 33. Finally, Thomas (2008) noted a similar pattern of progression through strategy type in their participants (obese adults who had attempted to lose weight): participants began by looking up and following diets they found in magazines as teenagers, then moved on to behavioural weight management programmes, and then turned to medications and diet supplements 51.

Differences in strategy use over time can also be observed through comparing those studies conducted exclusively in people attempting to achieve initial weight loss versus those conducted exclusively in people attempting to maintain weight loss. Generally, a wider range of strategies were discussed in relation to weight loss maintenance than in relation to acute weight loss attempts. In particular, weight loss maintenance narratives included more discussion of flexible restraint, goal setting, impulse management: awareness of motives, motivation, planning content, rule setting, self‐monitoring and stimulus control. In contrast, those studies focussing on weight loss included discussion of imitation (modelling) strategies, which did not arise in studies focussing exclusively on weight loss maintenance.

## Discussion

The most commonly discussed strategies involved restrictions, self‐monitoring, scheduling, professional support and weight management aids. With the exception of scheduling, for which participant experiences were predominantly positive, participants' attitudes and beliefs surrounding implementation of these strategies were mixed. Studies suggested that choice and use of these strategies changed throughout different stages of weight loss attempts, with a wider range of strategies discussed in relation to weight loss maintenance than to weight loss itself. The process of inductive coding in this review led to the expansion of the OxFAB taxonomy, with two new domains added, namely reframing and self‐experimentation.

To our knowledge, this is the first systematic review of qualitative studies to examine self‐directed weight loss efforts. Other qualitative reviews of weight loss in overweight and obese adults have included studies focussing on participant experiences of particular weight loss programmes 54, 55, 56, 57. Alhough these can be used to inform intervention development, the majority of adults currently trying to lose weight are doing so without the help of a formal programme, and therefore it is crucial we increase our understanding of this area. It is unsurprising that dietary restrictions and self‐monitoring were frequently the focus of the studies included in this review. Many weight loss interventions include these components 3, 9 and observational studies have linked them with improved weight loss and maintenance trajectories 58, 59, 60. In contrast, the other commonly mentioned strategies emerging from this review are less evident in interventions: in a recent systematic review of self‐help interventions for weight loss, only six of the 39 interventions recommended scheduling of diet and physical activity, and only two recommended weight management aids (Fig. 1) 9. These results suggest that the strategies people are using in self‐directed weight loss attempts do not always mirror those being suggested in self‐help interventions. Further research into these potential disconnects is needed, especially given that results from the qualitative studies in this review are in line with a recent observational cohort study in adults trying to lose weight, which found the majority were employing scheduling techniques and weight management aids as part of their weight loss attempts 10.

A major limitation of this review is the scope and quality of the included studies. Alhough some were high quality, quality assessment raised issues for many of the studies, and a number of the included studies were unpublished theses. This affects our confidence in the overall validity and consistency of our findings, although the full transcripts, which were available alongside many of the unpublished theses, go some way to alleviate these concerns. The majority of studies were undertaken in the US, and the vast majority were undertaken in the developed world among participants of higher socioeconomic status. Given cultural variation in strategies used and barriers to self‐directed weight loss that represent an unequal burden on people of lower socioeconomic groups, further studies in more diverse populations are needed 61, 62. In addition, few of the included studies focussed explicitly on weight loss strategies, and therefore, little detail was available on attitudes and beliefs surrounding these strategies. Given the nature of the available data, it is difficult to determine if the content of the studies accurately reflect the experiences of the participants, or if the studies' results have been tailored based on the interests of the researchers. Despite this, the studies still yielded rich data on weight loss strategies, pointing to the prominence of techniques and methods in participants' accounts of their weight loss experiences.

The limitations with study quality described in the preceding texts point to five specific recommendations relating to the methods and reporting of future qualitative studies in this area, which are informed by the CASP tool used to assess the studies in this review. 15 Firstly, many of the quality assessment domains were judged to be ‘unclear’ simply because of a lack of sufficient detail with which to make a judgement. In some part, this may be due to constraints on word length in published articles; where this is the case, authors should be encouraged to make study protocols available either through online registries or as supplemental material accompanying journal articles. Publishing study protocols would also allow readers to more effectively judge the extent to which individual study findings were guided by researcher expectations and biases. The second issue relates to recruitment methods, and ensuring the method is appropriate to meet the aims of the research. For example, in this review some studies aimed to capture experiences from a diverse range of people but ended up drawing on a very homogenous group. Often, snowball sampling was employed; where a study aims to capture a diverse sample, other methods for recruitment may be required. Thirdly, the relationship between the researcher and participants must be considered – as explained in the CASP tool, this includes the researcher critically examining their own role in terms of potential bias and influence during formulation of research questions and data collection. Fifthly, in terms of the data analysis process, it should be clear how categories and themes were derived when using thematic analysis, how the data presented were selected from the original sample and to what extent contradictory data were taken into account. Sufficient data should be presented to support the conclusions of the authors.

Alhough implications for future research are relatively clear, implications for practice are less so. Currently, empirical evidence is limited in its ability to identify effective cognitive and behavioural strategies for self‐directed weight loss attempts. Research is underway to further explore this area, but in the meantime, this lack of empirical evidence means we are unable to say based on the results of this review if the disconnect between the strategies used by individuals in self‐directed weight loss attempts and those prescribed by self‐help interventions reflect the fact that individuals are using less effective strategies, or reflect omissions in the self‐help interventions currently being tested. What is clear from this review is that a wide range of strategies are employed in self‐directed weight management, with patterns of use appearing to change over time, and attitudes towards strategy implementation varying based on individual circumstances. This suggests there may not be a ‘one‐size‐fits‐all’ approach to cognitive and behavioural strategies in self‐directed weight loss attempts.

In summary, this review points to a number of future directions. Further high‐quality primary studies are needed to explore experiences of self‐directed weight loss in overweight and obese adults, with a particular focus on choosing and implementing cognitive and behavioural techniques and on recruiting more diverse samples. This review will be used to inform revisions to the OxFAB taxonomy, in particular highlighting the phenomenon of ‘reframing’, not currently prevalent in behaviour change literature or included in existing behaviour change taxonomies 8, 53. Finally, it is intended that this review will act as a database that will be regularly updated, allowing for domain specific papers to be developed that will enable richer and more detailed analysis to be undertaken than was possible in this overview paper. A fuller understanding of the cognitive and behavioural strategies used in self‐directed weight loss efforts has the potential to enrich the advice provided to individuals trying to lose weight on their own; at present, this review suggests that interventions targeting these individuals do not necessarily reflect the lived experience of self‐directed weight loss.

## Conflict of interest statement

No conflict of interest was declared.

## Supporting information


**Figure S1** PRISMA diagram of study flow.

Supporting info item
