# Supplementary material for: Cognitive and behavioural strategies for self‐directed weight loss: systematic review of qualitative studies
Source: Obes Rev. 2017 Jan 24;18(3):335–49. doi: 10.1111/obr.12500 (PMC5408390; doi:10.1111/obr.12500)

Cognitive and behavioural strategies for self-directed weight loss: systematic review of qualitative studies

Jamie Hartmann-Boyce, Anne-Marie Boylan, Susan A. Jebb, Ben Fletcher, Paul Aveyard
Nuffield Department of Primary Care Health Sciences, University of Oxford

Corresponding author: Jamie Hartmann-Boyce, Nuffield Department of Primary Care Health Sciences, University of Oxford, Radcliffe Observatory Quarter, OX2 6GG, UK. Email: Jamie.hartmann-boyce@phc.ox.ac.uk

Figure S1 PRISMA diagram of study flow

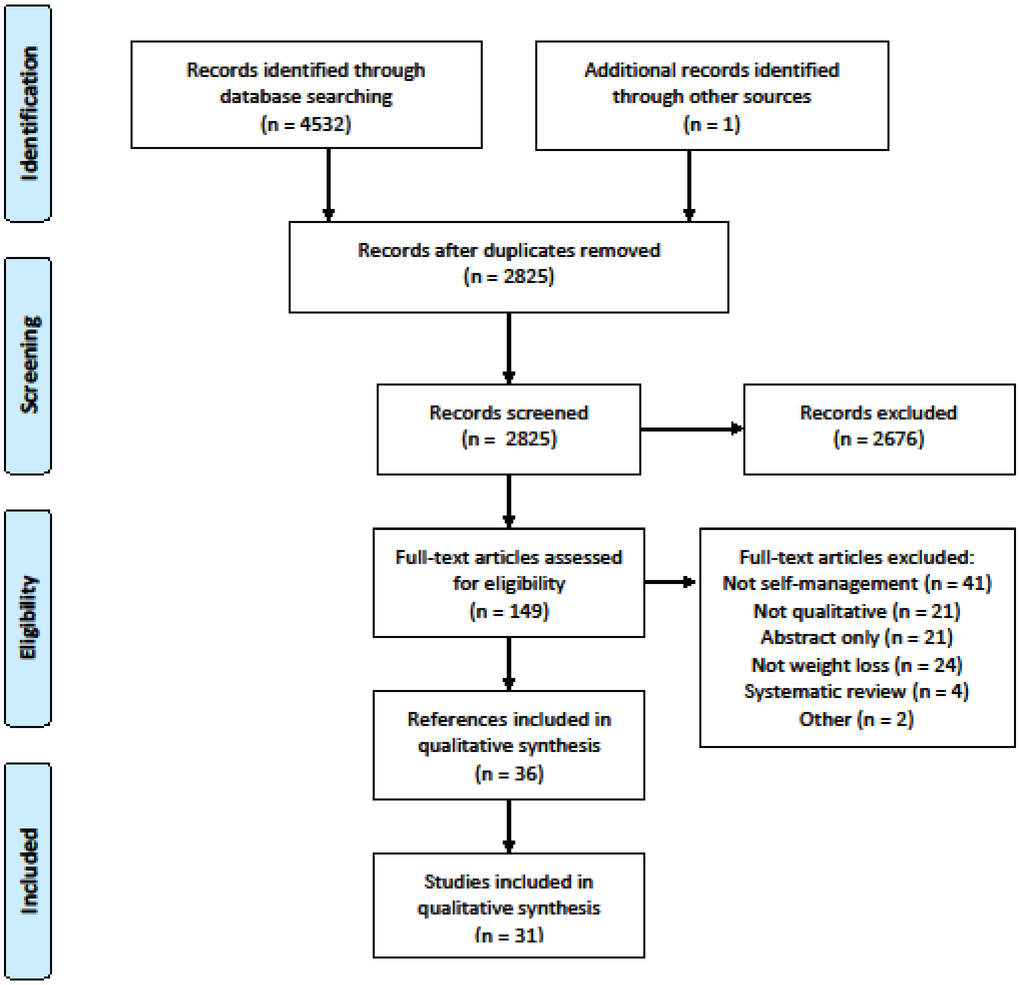

Supplement: Supplementary file 1 — Supporting info item [file OBR-18-335-s001.pdf]
